# Chloroplast genomes as a tool to resolve red algal phylogenies: a case study in the Nemaliales

**DOI:** 10.1186/s12862-016-0772-3

**Published:** 2016-10-10

**Authors:** Joana F. Costa, Showe-Mei Lin, Erasmo C. Macaya, Cindy Fernández-García, Heroen Verbruggen

**Affiliations:** 1 School of BioSciences, University of Melbourne, Parkville, VIC 3010 Australia; 2 Institute of Marine Biology, National Taiwan Ocean University, Keelung, 20224 Taiwan; 3 Departamento de Oceanografıa, Universidad de Concepción, Casilla, 160-C Chile; 4 Millennium Nucleus Ecology and Sustainable Management of Oceanic Island (ESMOI), Coquimbo, Chile; 5 Escuela de Biología, Centro de Investigación en Ciencias del Mar y Limnología (CIMAR), Universidad de Costa Rica, San Pedro, San José 11501-2060 Costa Rica

**Keywords:** Plastid genomes, Chloroplast phylogenomics, Red algae, Nemaliales, Conserved genomes

## Abstract

**Background:**

Obtaining strongly supported phylogenies that permit confident taxonomic and evolutionary interpretations has been a challenge in algal biology. High-throughput sequencing has improved the capacity to generate data and yields more informative datasets. We sequenced and analysed the chloroplast genomes of 22 species of the order Nemaliales as a case study in the use of phylogenomics as an approach to achieve well-supported phylogenies of red algae.

**Results:**

Chloroplast genomes of the order Nemaliales are highly conserved, gene-dense and completely syntenic with very few cases of gene loss. Our ML estimation based on 195 genes recovered a completely supported phylogeny, permitting re-classification of the order at various taxonomic levels. Six families are recognised and the placement of several previously contradictory clades is resolved. Two new sub-orders are described, Galaxaurineae and Nemaliineae, based on the early-branching nature and monophyly of the groups, and presence or absence of a pericarp. Analyses of subsets of the data showed that >90 % bootstrap support can be achieved with datasets as small as 2500 nt and that fast and medium evolving genes perform much better when it comes to resolving phylogenetic relationships.

**Conclusions:**

In this study we show that phylogenomics is an efficient and effective approach to investigate phylogenetic relationships. The six currently circumscribed Nemaliales families are clustered into two evolutionary lineages with strong statistical support based on chloroplast phylogenomic analyses. The conserved nature of red algal chloroplast genomes is a convenient and accessible source of data to resolve their ancient relationships.

**Electronic supplementary material:**

The online version of this article (doi:10.1186/s12862-016-0772-3) contains supplementary material, which is available to authorized users.

## Background

Molecular phylogenies are the cornerstone of biodiversity and evolutionary research but many phylogenetic relationships are contradictory or not known with certainty, for example due to low statistical support. One of the major challenges in designing phylogenetic studies is to decide how much molecular data is needed to achieve a satisfactory result [1, 2].

High throughout sequencing (HTS) techniques have made the acquisition of multilocus datasets easy, even for non-model organisms. Their use has become common practice for comparative and phylogenetic analyses of entire genomes (phylogenomics). This provides us with insights not only into phylogenetic relationships but also how other features of the genome (e.g. genome synteny, gene loss, intergenic regions) evolve. While obtaining eukaryotic nuclear genomes still presents significant challenges in terms of sequencing coverage, assembly and annotation [3], for photosynthetic organisms there is a more accessible alternative - their plastid genomes.

Chloroplast genomes are an attractive option for phylogenomic studies for various reasons. First of all, they are present in multiple copies in each cell, therefore chloroplast DNA (cpDNA) data is easily obtained from bulk DNA extractions. Additionally, they are relatively small in size (~100-190 kb) and exhibit low variability in gene content and (in some groups) gene arrangement [4], meaning that assembly and annotation are straightforward. Furthermore, the non-recombinant nature of plastids makes them a good tool when inferring ancient phylogenetic relationships [5].

The oldest eukaryotic fossil is believed to be a red alga (*Bangiomorpha pubescens*) dated as being 1.2 billion years old [6]. However resolving the red algal tree of life has been challenging with the relationships between Florideophyceae – the most diverse class of red algae – especially difficult to resolve. While the five florideophyte subclasses are well supported, within these lineages many early-branching nodes are yet to be resolved [1, 7, 8]. A study comparing ten red algae chloroplast genomes showed the potential of plastid phylogenomics to unravel relationships among red algal classes and their constituent lineages [9]. Among the eukaryotes with primary plastids (Archaeplastida), red algal chloroplast genomes are the most conserved and have the highest gene content [9, 10]. This is a potential perfect combination of features to resolve the ancient relationships among red algal groups: a conserved architecture of the genome, which simplifies data processing, and a high number of genes that are likely to hold enough phylogenetic signal.

A persistent problem within the Florideophyceae is found in the order Nemaliales. The Nemaliales belong to the subclass Nemaliophycideae, one of the earliest branching clades within the florideophytes. Molecular clocks suggest that the Nemaliales diverged from other Nemaliophycideae lineages approximately 200 Ma ago [8]. There are 276 species of Nemaliales currently described [11] distributed across 34 genera and 6 families. The Liagoraceae is the most species-rich family, followed by the Scinaiaceae and Galaxauraceae. Three monogeneric families have been recently recognised: the re-instated Nemaliaceae and the new Yamadaellaceae and Liagoropsidaceae [12]. However, the phylogenetic relationships among these six families have not been resolved with confidence and we lack a comprehensive reference phylogeny for the group. The placement of Scinaiaceae has been contradictory in previous studies [12–14] and the relationships between the remaining families have low support [12]. The early branches within the Liagoraceae also have low support [12, 15].

Analyses of whole cpDNA genomes greatly improved phylogenetic resolution in the green plant lineage [16–20]. Despite its promising features, chloroplast phylogenomics has not been widely applied to resolve phylogenetic relationships among red algae. The Nemaliales are a good model to assess the utility of plastid phylogenomics in red algae - an old photosynthetic group with ambiguous phylogenetic relationships.

This study aims to (1) characterize chloroplast genomes of Nemaliales, (2) use the data to reconstruct a well-supported phylogenetic tree and, based on these results, (3) revise the classification of the order and (4) evaluate the utility of chloroplast phylogenomics in red algae. Our approach consists of high-throughput sequencing of a phylogenetically diverse set of Nemaliales species, comparison of genomes across the group, and phylogenetic analyses of the complete dataset and subsets thereof.

## Methods

### Taxon sampling and sequencing

We selected 19 taxa representing the six families of Nemaliales and 3 closely related outgroup species from the orders Palmariales and Acrochaetiales (Table 1).

**Table 1:** Summary of chloroplast genome statistics for Nemaliales and outgroups (Palmariaceae, Rhodothamniellaceae, Acrochaetiaceae). All specimen vouchers are deposited at MELU herbarium

Family	Species	Acession #	Specimen ID	Origin	Genome size (BP)	GC%	Protein coding genes	ORFs	tRNA	tmRNA	rRNA	ncRNA	introns
Scinaiaceae	*Nothogenia fastigiata*	SAMEA4358435	J.0141	Chile	182 457	33.0	193	8	30	1	3	1	2
	*Scinaia undulata*	SAMEA4478602	J.0081	Chile	183 795	35.9	197	9	31	1	3	1	2
Galaxauraceae	*Actinotrichia fragilis*	SAMEA4357171	HV04073	Philippines	183 324	29.8	197	9	31	1	3	1	2
	*Galaxaura rugosa*	SAMEA4357173	JFC0074	Australia	181 215	29.6	197	7	31	1	3	1	2
	*Tricleocarpa cylindrica*	SAMEA4478608	J.0145	Costa Rica	≥150 119	-	≥149	≥8	≥25	≥1	≥1	≥1	≥0
	*Dichotomaria marginata*	SAMEA4357172	HV04060	Philippines	184 395	28.8	197	11	31	1	3	1	2
Liagoraceae	*Liagora brachyclada*	SAMEA4395348	J.0126	Chile	182 937	33.7	195	9	31	1	3	1	2
	*Liagora harveyana*	SAMEA4358432	J.0237	Chile	182 933	33.9	195	10	31	1	3	1	2
	*Izziella formosana*	SAMEA4358392	J.0158	Costa Rica	183 248	35.0	195	9	31	1	3	1	2
	*Neoizziella asiatica*	SAMEA4358434	J.0154	Costa Rica	183 313	33.4	195	10	31	1	3	1	2
	*Titanophycus setchellii*	SAMEA4478603	J.0604	Japan	≥183 356	-	195	≥9	≥31	≥1	≥3	≥1	≥2
	*Helminthora furcellata*	SAMEA4393237	J.0165	South Africa	184 585	32.1	195	8	31	1	3	1	2
	*Trichogloeopsis pedicellata*	SAMEA4358437	C.0024	French West Indies	183 497	31.9	194	8	31	1	3	1	2
	*Hommersandiophycus borowitzkae*	SAMEA4358391	HV00480	Jamaica	184 728	32.2	194	9	31	1	3	1	2
	*Dermonema virens*	SAMEA4357169	J.0258	Taiwan	184 997	34.1	195	9	31	1	3	1	2
	*Helminthocladia australis*	SAMEA4358292	J.0167	South Africa	185 694	32.8	195	9	31	1	3	1	2
Yamadallaceae	*Yamadaella caenomyce*	SAMEA4358436	J.0255	Chile	182 460	35.9	194	9	31	1	3	1	2
Liagoropsidaceae	*Liagoropsis maxima*	SAMEA4358433	J.0256	Taiwan	189 564	32.1	195	11	31	1	3	1	2
Nemaliaceae	*Nemalion* sp.	SAMEA4478604	H.1444	Italy	182 930	35.5	194	7	31	1	3	1	2
Palmariaceae	*Palmaria palmata*	SAMEA4478605	ODC1024	France	≥187 103	-	≥196	≥11	≥31	≥1	≥3	≥1	≥2
Rhodothamniellaceae	*Rhodothamniella floridula*	SAMEA4478606	HEC15602	France	≥182 494	-	≥196	≥9	≥29	≥1	≥1	≥1	≥2
Acrochaetiaceae	*Acrochaetium secundatum*	SAMEA4393238	FS1067	Bulgaria	≥183541	-	≥196	≥12	≥29	≥1	≥0	≥1	≥2

Genomic DNA was isolated from silica gel dried tissue or herbarium vouchers using an adapted CTAB protocol [21]. In brief, samples were incubated at 60 °C for an hour in CTAB buffer with proteinase K and DNA was extracted in two subsequent steps with 24:1 chloroform:isoamyl alcohol. DNA was precipitated in 80 % isopropanol at 4 °C for 2 h and eluted in 0.1× TE buffer.

Library preparation and sequencing was performed either at the Georgia Genome Facility (University of Georgia, GA, USA) or at the Genome Center of the Cold Spring Harbor Marine Laboratory (NY, USA) using different Illumina platforms (Additional file 1). For the first sequencing run, libraries of 350 nucleotide (nt) fragments were prepared from DNA extracts of each sample using a TruSeq Nano LT kit. Each library was given a unique barcode and sequenced on the Illumina HiSeq 2000 platform. Because the laboratory at the University of Melbourne is carrying out chloroplast genome projects of green and red algae, for subsequent runs we pooled DNA extracts of a red and a green alga, resulting in substantial savings for library preparation. For these, libraries of 500 nt fragments were prepared using a KAPABIOSYSTEMS DNA Library Preparation Kit (KK8232) and sequenced on either HiSeq 2500 or NextSeq 500.

### Assembly, annotation and synteny

Assembly and annotation followed [22]. In brief, original sequencing reads were trimmed with CLC Genomics Workbench 7.5.1 (CLC bio, Aaarhus, Denmark) with a quality threshold of 0.05 and de novo assembly was performed in both CLC Genomics Workbench 7.5.1 and MEGAHIT v0.1.2 [23]. In CLC, assembly was performed with automatic k-mer size and default parameters. In MEGAHIT, we used 10 kmer sizes (21–91 in steps of 10 and 99).

Chloroplast contig sequences were identified with blastx searches against a custom-built database containing known plastid genes of Florideophyceae. Contigs identified as cpDNA from Nemaliales were imported to Geneious 6.1.7 where any ambiguities were resolved by mapping original reads (medium sensitivity, up to 5 iterations). Different assemblers gave similar results, with contigs often having a different starting position in the genome. The circular-mapping nature of the genomes was predicted by mapping the end and start (~1000 bp) of a given contig to the contig inferred by the other assembler.

Gene prediction was carried out in MFannot (http://megasun.bch.umontreal.ca/cgi-bin/mfannot/mfannotInterface.pl) and Glimmer 3 [24], and manually inspected and annotated in Geneious 6.1.7. Considering the collinearity of red algae chloroplast genomes, visual inspection of contigs was performed simultaneously across multiple species to help identify unrecognised or misidentified genes by the automated tools. Colinearity became particularly useful for undetected genes. If the automated tools did not identify a gene in a given species, we would align that gene from all other species with the genome region where we would expect to find it. Depending on levels of similarity, alignment quality and the presence of start and stop codons, the gene was either annotated or not. Once contigs were completely annotated, coding sequences were extracted and gene alignments across species were built. These alignments were manually verified and the procedure was repeated until all remaining annotation issues (e.g. positions of start codons) were resolved.

Synteny between genomes was compared using the progressive Mauve algorithm in Geneious 6.1.7 using the full alignment option, automatically calculated seed weights and automatically calculated minimum locally collinear blocks (LCB) score.

### Phylogenetic analysis

We filtered our final gene alignments to retain only CDSs (coding DNA sequences) present in more than 6 taxa. Alignments of individual genes were performed at the amino acid level using MAFFT v7.245 [25] and nucleotide alignments recovered based on the matching amino-acid alignment using revTrans [26]. Both amino-acid (aa) and nucleotide alignments (nt) were checked manually in Geneious and concatenated. For both the nt and aa alignments, phylogenetic trees were estimated using maximum likelihood (ML) with RAxML v8.0.26 [27]. We analysed both datasets from 500 randomized maximum parsimony starting trees with (1) a model suggested by a model tester (see below), and (2) a much simpler model to test if model choice would affect topology and bootstrap support. For the aa data, the ML phylogeny was inferred using both a cpREV + Γ + F model as suggested by ProtTest 3.4.1 [28] and a simple LG + CAT model. For the nt data, estimations were done using a simple GTR + Γ model and a partitioned GTR + Γ + I model as suggested by PartitionFinder [29].

### Data requirement simulations

In order to evaluate how much chloroplast genome data are needed to resolve the phylogeny and whether genes with different rates of evolution performed better or worse at resolving the phylogeny, we analysed subsets of the data and evaluated how support changed as a function of the size of the subset.

First, we calculated the rates of individual genes. We optimized a GTR + Γ model using the ML tree obtained from the concatenated dataset and re-calculated the branch lengths from the gene alignments. The total tree length was than divided by the length of the tree obtained from the concatenated alignment. This value reflects the rate of the gene relative to the overall rate of the protein-coding parts of the chloroplast genome, with genes >1 being faster and genes <1 slower.

Second, we performed analyses on random subsets of the alignment. For this, non-parametric bootstrapping was used to create datasets of different sizes (1 k, 2.5 k, 5 k, 10 k, 25 k, 50 k, 100 k) and those datasets were analysed using RAxML with a GTR + Γ model. The bootstrap support was summarized and plotted as a function of alignment size. Five replicate analyses each consisting of 100 bootstrapped datasets were run for each alignment size. This procedure is similar to that used by Verbruggen et al. [1] but without extrapolation beyond the original alignment size.

Third, we extended the non-parametric bootstrapping method to evaluate how well fast, medium rate and slow genes performed in terms of inferring the phylogeny. Genes less than 800 nt were excluded for these analyses. We subdivided the remaining genes into slow, medium rate, and fast categories (using relative rate thresholds of 0.75 and 1.5). Then, genes were concatenated within each category and the procedure from the paragraph above was repeated. Alignment lengths were kept shorter (1 k, 2.5 k, 5 k, 10 k) because fewer data are available per category and after exclusion of short genes.

## Results

### Sequencing

To resolve the Nemaliales phylogeny we assembled and characterised 17 complete and 5 draft plastid genomes. The average coverage of the plastid contigs varied between 130× in one of the *Tricleocarpa* contigs and 4,140× in *Izziella* (see Additional file 1 for sample by sample coverage). Overall samples sequenced with the NextSeq500 presented better coverage (Additional file 1). We are uncertain about the reason behind this, it could be for a number of reasons, for example, because more data was generated or because assemblers perform better with longer reads.

### Assembly

Of the plastid genomes we consider complete, all but one assembled into a single contig using the automatic assemblers. The exception (*Actinotrichia*) assembled into three contigs that showed similarity to Nemaliales’ plastid DNA. The contigs overlapped in two different positions by 892 bp and 268 bp and could be manually joined into a supercontig, and read mapping showed good coverage across the joints. Circularity could be confirmed for the supercontig and it was collinear to complete genomes of the closely related *Dichotomaria* and *Galaxaura*.

For the ingroups *Tricleocarpa* and *Titanophycus* as well as the three outgroups, chloroplast genomes were assembled into multiple contigs. We were able to join the multiple contigs manually for the three outgroups and *Titanophycus* but were unable to confirm their circularity. Extending the original contigs through read mapping did not result in matching ends due to ambiguities in the assembly of the ribosomal RNA cistron (rns/rnl region). Nonetheless, all genes identified in the circular mapping genomes were also present in these four draft genomes. For *Tricleocarpa*, contigs were short (6628 bp – 79,809 bp) and they could not be joined with confidence.

### Genome structure

Members of the Nemaliales, Palmariales and Acrochaetiales have large, gene-dense and highly conserved chloroplast genomes. The shortest plastid in the Nemaliales was found in *Galaxaura* and the longest in *Liagoropsis* (Table 1). This difference (8349 bp) is in part due to variation in the length of genes as well as intergenic spacers. In *Liagoropsis*, for instance, genes are 3.5 bp longer on average and spacers 15 bp longer than in the other Nemaliales.

**Fig. 1:**
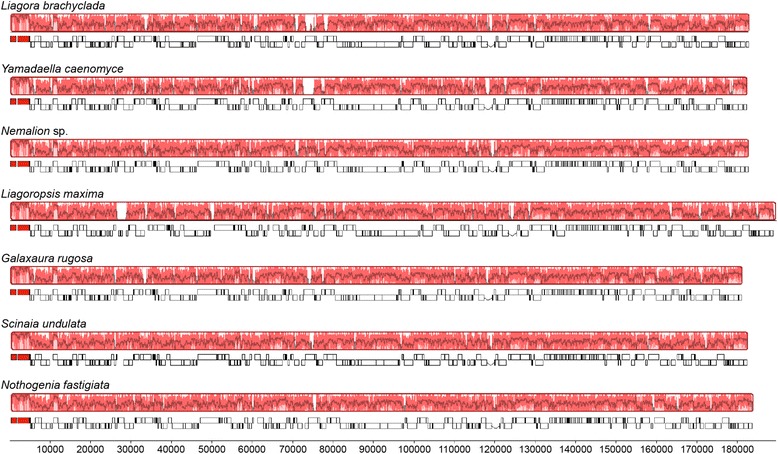
Nemaliales plastid genomes alignment. Mauve alignment showing the conserved structure of chloroplast genomes between the six Nemaliales families (Liagoraceae – *L. brachyclada*; Yamadaellaceae – *Y. caenomyce*; Nemaliaceae – *Nemalion* sp.; Liagoropsidaceae – *L. maxima*; Galaxauraceae – *G. rugosa*; Scinaiaceae – *S. undulata* and *N. fastigiata*). Horizontal axis refers to genome length in bp. Synteny between genomes is represented by Locally Collinear Blocks (LCB). Within each LCB a sequence similarity profile is shown. Gene maps are represented below LCBs, protein coding genes in white, rRNA genes in red, position of boxes above or below line refers to gene orientation

Despite some minor differences in gene content (described below), the overall architecture of Nemaliales chloroplast genomes suggests that there have been no rearrangement or inversion events (Fig. 1). The Mauve alignment shows a single local collinear block (LCB), revealing that cp genomes are completely syntenic across the entire order. Plastid genes were found to be in exactly the same positions and directions, and nucleotide sequences presented high levels of similarity, varying between 45.8 and 91 %.

### Gene content and introns

Most Nemaliales have 195 plastid protein-coding genes, 31 tRNAs, 1 tmRNA, 3 rRNAs and 2 introns. Protein coding genes varied between 193 in *Nothogenia* and 197 in Galaxauraceae and *Scinaia*. The difference is due to occasional gene losses.

The most widely lost gene is *pbs*A. It is absent in *Nothogenia*, *Trichogloeopsis*, *Nemalion* and *Yamadaella*. The hypothetical protein *ycf41* is absent in *Hommersandiophycus* and *petP* (*ycf86*) was only found in the Galaxauraceae family and in *Scinaia*. The hypothetical proteins *ycf35* and *ycf46* in *Nothogenia* appear to be pseudogenes having similar aa sequences but premature stop codons. The same pseudogene pattern is found for *ycf21* in Yamadaellaceae, Liagoropsidaceae and Liagoraceae. tRNA-*Met* is present in all taxa in 3 copies.

We found two group II introns in all taxa. The one in the *chlB* gene and contains an intronic ORF. The second group II intron was found in one of the tRNA-*Met* copies.

### Alignment statistics

The alignment of concatenated amino acid sequences comprised 48,470 characters of which 23,152 were variable (47.7 %) and 18,454 (79.7 %) parsimony-informative. The concatenated nucleotide alignment is 145,410 bp long, with 86,313 variable sites (59.3 %) of which 76,031 (88 %) were informative.

### Phylogeny

**Fig. 2:**
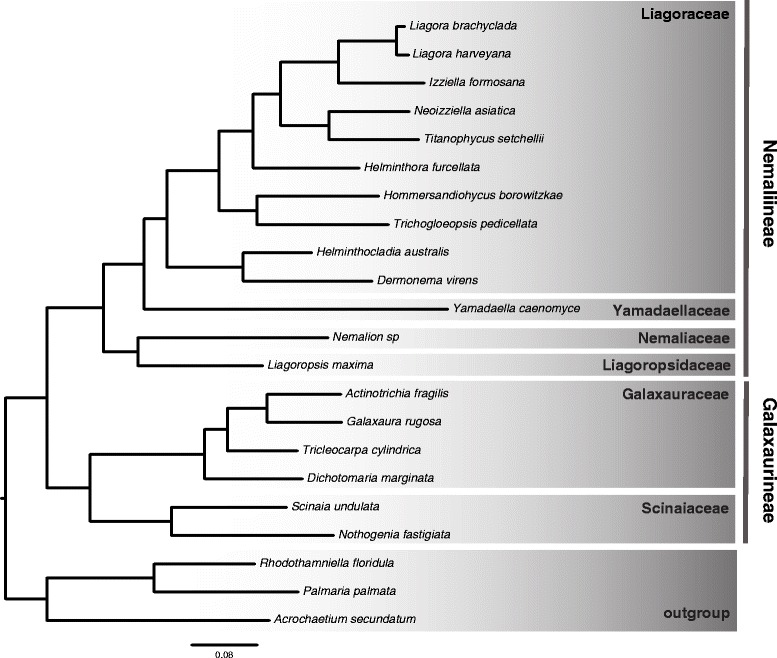
Phylogenetic tree of Nemaliales with the new proposed sub-orders (Nemaliineae and Galaxaurineae). Maximum-likelihood estimated tree based on protein alignment of 195 concatenated genes showing resolved inter-family relationships. 100 bootstrap support was recovered for every branch. A tree inferred from corresponding nucleotide data (not shown) is identical in topology and support

The ML phylogenetic trees inferred from nucleotide and amino acid datasets are identical and both present perfect bootstrap support at all nodes (Fig. 2). Model choice also did not affect the topology or support. The earliest bifurcation in the ingroup separates Galaxauraceae and Scinaiaceae from the Nemaliaceae, Liagoropsidaceae, Yamadaellaceae and Liagoraceae. Within the Galaxauraceae, *Dichotomaria* is the earliest branching genus and *Actinotrichia* and *Galaxaura* are recovered as sister lineages. Nemaliaceae and Liagoropsidaceae are shown to be sister families, and the same is true of Yamadaellaceae and Liagoraceae. Within the Liagoraceae, *Helminthocladia* and *Dermonema* form an early-branching clade. *Hommersandiophycus* clusters with *Trichogloeopsis*. The sister genera *Titanophycus* and *Neoizziella* are the most closely related to the *Liagora* + *Izziella* clade.

### Data requirement simulations

**Fig. 3:**
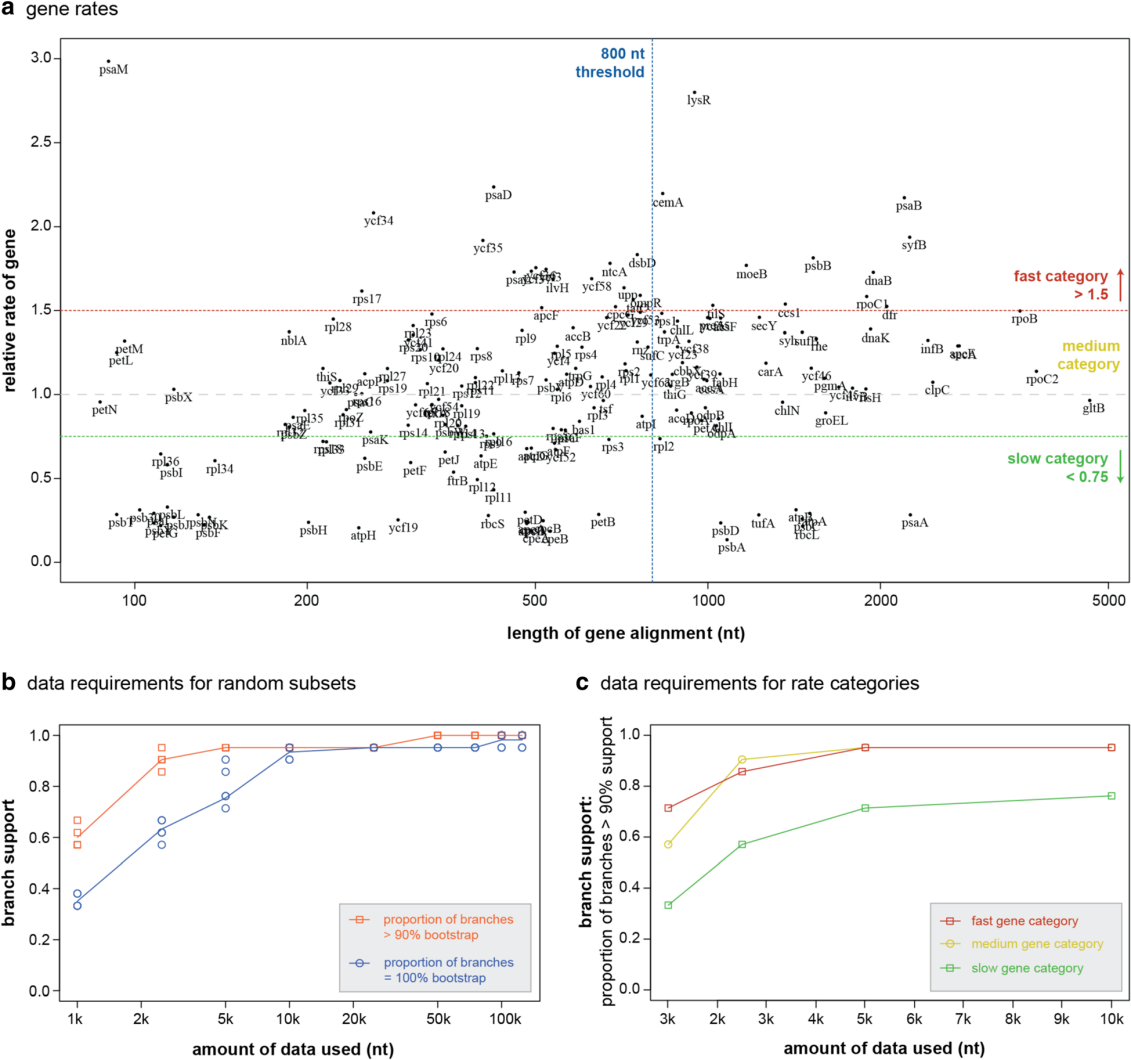
Data requirement simulations. **a** Gene rates categories. Annotated chloroplast genes divided into rate categories: slow, medium and fast evolving genes. Only genes in alignments bigger than 800 nt (*blue line*) were considered further. **b** Data requirements derived from analyses of subsets of data. Graph shows data required to achieve both >90 % (*orange*) and 100 % (*blue*) bootstrap support from random gene alignments. **c** Data requirement by rate categories. Bootstrap support >90 % in relation to the amount of data needed for the gene rates categories. Medium (*yellow*) and fast (*red*) evolving genes reached high support with significantly less data than slow evolving genes (*green*)

Figure 3a shows that the relative rates of chloroplast genes vary over an order of magnitude, with the slowest genes including those commonly used for molecular phylogenetics (*rbc*L, *psa*A, *psb*A, *psb*D, *psb*C, *atp*A, *atp*B, *tuf*A). The great majority of genes, however, are substantially faster than these. We have subdivided the figure into three vertical partitions to define slow, medium and fast categories of genes using arbitrary thresholds of 0.75 and 1.5. The original data for this figure can be consulted as online supporting material (Additional file 2).

Random subsampling of our entire chloroplast alignment showed that analyses with 2500 nt already achieved a high proportion of branches exceeding 90 % bootstrap support (Fig. 3b). With alignments of 10,000 nt and up, the great majority of branches had reached 100 % bootstrap support (Fig. 3b).

A similar analysis carried out on fast, medium-rate and slow genes showed that while fast and medium-rate genes rapidly achieve high bootstrap support, slow genes ramp up much more slowly (Fig. 3c). It is striking that even for alignments with 10,000 nt from slow genes, bootstrap support is inferior to alignments of just 2500 nt from fast or medium-rate genes. Trees inferred from concatenated alignments of the three rate categories recovered the same topology.

## Discussion

### Nemaliales plastid genomes

Chloroplast genomes in the order Nemaliales are consistent with those of other red algae: they are large, gene-dense and feature a highly conserved gene order. The longest known plastid genome in red algae are found in the Bangiophyceae with the longest being *Porphyra pulchra* with ~194 kb followed by the coralline *Sporolithon durum* with ~191 kb [10]. *Liagoropsis* is the third longest plastid genome described so far with ~189 kb. The difference in plastid size within the order is only ~8000 bp, and even the smallest among them (*Galaxaura*) still rank among the largest plastid genomes of Archaeplastida [4].

Within Nemaliales families, the earliest divergent clades typically exhibit longer plastid genomes. However, this is not a result of differing gene content. Gene composition is highly conserved with only five genes absent in some of the taxa. The differences in genome sizes are caused mostly by changes in the length of non-coding regions. For example, within Liagoraceae, the *Liagora* lineage has reduced intergenic spacers when compared to *Helminthocladia* or *Dermonema*; the same is true within Galaxauraceae, where the lineage *Actinotrichia* + *Galaxaura* present shorter intergenic regions when compared to *Dichotomaria* (Additional file 1). This pattern could be of evolutionary interest in the light of the ‘lower-cost strategy’ [30] and faster replication of plastids [31].

The highly conserved genome architecture in Nemaliales is remarkable considering that the order diverged ~200 Ma ago [8] and other groups of algae show much higher levels of rearrangement [32, 33].

The only gene missing from several Nemaliales taxa is *pbsA*, an important gene involved in the production of phycobilins, the light-harvesting proteins characteristic of red algae [34]. This gene has been lost in several taxa in the Rhodomelaceae as well (P. Diaz Tapia, personal communication) but other than that has never been reported missing from florideophyte plastids. In the unicelular Cyanidiophyceae, the earliest divergent class of red algae, it has been found in the nucleus instead [35, 36] but phylogenetic inferences showed that this nuclear *pbsA* gene is more closely related to nuclear *pbs*A from green plant lineage than to plastid *pbs*A of other red algae [34]. This suggests that the Cyanidiophyceae nuclear *pbsA* gene was not transferred from the plastid. We cannot confirm whether *pbsA* has been transferred to the nucleus in Nemaliales.

*petP* is a subunit of the cytochrome *b*_*6*_ *f* complex with no essential role in the overall function of the complex [37]. It has been identified in cyanobacteria and in bangiophytes but it is apparently absent in green algae and land plants [38]. It is found in *Chondrus* and *Grateloupia* [9, 39], and in this study in the Galaxauraceae and *Scinaia*. This suggests that it might have been lost twice within the Nemaliales: once in *Nothogenia* and once in the ancestor of the Nemaliaceae + Liagoropsidaceae + Yamadaellaceae + Liagoraceae clade.

The third missing gene, absent in *Hommersandiophycus*, is *ycf41. Ycf41* is a hypothetical protein of unknown function that has been annotated in heterokonts [40] and in all florideophytes’ plastid genomes with the exception of *Laurencia* in the Rhodomelaceae [22].

Three pseudogenes are found in the Nemaliales. The function of *ycf21* is unknown and it was also found as a pseudogene in *Sporolithon durum* [10]. The function of *ycf35* and *ycf46* genes is still uncertain but they are thought to be involved in CO_2_ uptake and utilization [41]. These genes are present in cyanobacteria and other eukaryotic algae but have been lost in green plants [40–43]. Within the florideophytes they have only been reported absent in the coralline *Calliarthron* [9]*.*

Gene loss in red algal plastids is rare and there is no apparent functional relationship between genes being lost, suggesting it is a stochastic process. Apart from *pbsA*, the genes not found in some Nemaliales taxa are either of unknown function or play no essential role.

Red algal plastids present an uncommonly low number of introns [9, 10] and only two group II introns were found in the Nemaliales. Group II introns are found in bacterial, chloroplast and mitochondrial genomes, are self-splicing and are believed to be the ancestor of spliceosomal introns [44, 45], which are considered to be crucial in the evolution of eukaryotes [46]. One of these introns was found in the tRNA-Met gene in all taxa. This intron is present in all other florideophyte plastids described to date [9, 22] but not for the classes Cyanidiophyceae, Porphyridiophyceae and Bangiophyceae [47–49]. Despite being uncommon in plastid tRNAs, group II introns appear to be retained once they are acquired [50] which suggests that this intron was gained by a Florideophyceae ancestor. A second group II intron was found in the *chlB* gene that together with *chlN* and *chlL* encodes for the LIPOR enzyme responsible for chlorophyll synthesis in the absence of light [51]. This gene complex has been lost extensively across different photosynthetic eukaryotic lineages with no evidence of it being transferred to the nucleus [52]. It was also widely lost in florideophytes. They were only reported in this study and in the Corallinales [9, 10]. Following the divergence of Hildenbrandiophyceae, the Coralinales and Nemaliales are the earliest divergent clades in the Florideophyceae suggesting that the loss of the LIPOR complex happened later in the evolution of the group. These three lineages also happen to be the only calcified florideophytes, which might explain the preservation of a complex for light-independent chlorophyll synthesis. However, if the LIPOR complex is present, always has this group II intron in *chlB* gene, that along with the tRNA-Met intron, could have been acquired when the florideophytes diverged from the other red algal lineages.

It is still not understood which phenomena are causing such high level of conservatism in red plastids. It seems likely that most of the genes found in the plastid across all red algae are essential for plastid function, but in other algal lineages part of these genes have been transferred to the nucleus.

### Phylogeny of Nemaliales

Based on a diverse set of Nemaliales, our cp phylogenomics approach recovered a fully resolved phylogeny of the order that was previously unsupported [12, 13, 15, 53, 54].

Our phylogeny confirms that the Nemaliales is comprised of six families. Scinaiaceae, Galaxauraceae and Liagoraceae are long-recognised families [13] and the early branching nature of the recently resurrected Nemaliaceae and the newly proposed Yamadaellaceae and Liagoropsidaceae supports their recognition at a high taxonomic level.

The three new families were proposed based on a concatenated dataset of *psaA* and *rbcL* [12]. Our larger dataset improves the resolution at deep nodes thus we can confidently confirm their relationships. The Yamadaellaceae and Liagoropsidaceae develop multiple initial gonimoblasts per zygote, whereas in the Liagoraceae and Nemaliaceae a single one is produced [12]. Considering that Galaxauraceae and Scinaiaceae [53, 55] zygotes produce a single initial gonimoblast it is likely that this was the ancestral state and that the production of multiple gonimoblasts was independently acquired by Yamadaellaceae and Liagoropsidaceae.

The placement of Scinaiaceae has been a controversial node in phylogenetic studies [12–14]. It is now clear that this exclusively non-calcified family is more closely related to the exclusively calcified Galaxauraceae than to any other family.

This phylogenomics approach also revealed to be useful at lower taxonomic levels. Within Galaxauraceae, *Dichotomaria* is the earliest diverging genus and *Tricleocarpa* position within the family is finally resolved showing that *Actinotrichia* and *Galaxaura* are sister lineages. These relationships were contradictory and had low support in recent multi-gene phylogenies [14, 56].

In Liagoraceae, the overall topology of our ML tree is mostly concordant to recent phylogenies of the family of both plastid and nuclear markers [12, 15] but where previously support was low to weak, our tree is resolved at all levels. The non-calcified *Helminthocladia* and *Dermonema* form the earliest divergent clade within the Liagoraceae. It is interesting to consider that these deeper branching genera were once accepted as the type genus’ of two distinct families: Helminthocladiaceae [57] and Dermonemataceae [58], respectively. As previously shown, the newly described genus *Hommersandiohycus* [59] clusters with *Trichogloeopsis. Helminthora* in our tree represents a lineage of its own but previous studies have shown that it clusters with high support with *Cumagloia* [12, 59, 60], a genus not included in our analysis. Curiously, *Liagora*, the generitype and the most diverse genus in the family diverged later within the clade.

### Re-classification of Nemaliales

The fact that our ML inference is fully supported across all taxonomic levels allows us to propose a more refined classification with high confidence. Therefore we suggest taxonomic rearrangement of the Nemaliales order to be composed of two sub-orders: the Galaxaurineae for the Galaxauraceae and Scinaiaceae and the Nemaliineae for the remaining families.

**Galaxaurineae** J.F. Costa, S.M. Lin, E.C. Macaya, C. Fernández-García, H. Verbruggen **subordo nov**.

Diagnosis: Recognized primarily based on monophyly in phylogenetic trees derived from chloroplast genome data. Members form a single intial gonimoblast from the zygote, and carposporophytes are immersed in conceptacles covered with a consolidated pericarp. Two families, Galaxauraceae and Scinaiaceae, are included. The morphological features used for separating genera mainly including a combination of cortex morphology, the heteromorphic, dimorphic or isomorphic life history whether or not involucral/sterile filaments intermixing with gonimoblasts.

### Genera included in suborder

Galaxauraceae

- ***Galaxaura*** **Lamouroux (type genus)**
- *Actinotrichia* Decaisne
- *Dichotomaria* Lamarck

Scinaiaceae

- *Scinaia* Bivona-Bernardi
- *Tricleocarpa* Huisman & Borowitzka
- *Nothogenia* Montagne
- *Gloiophloea* Agardh
- *Whidbeyella* Setchell & N. L. Gardner

**Nemaliineae** J.F. Costa, S.M. Lin, E.C. Macaya, C. Fernández-García, H. Verbruggen **subordo nov**.

Diagnosis: Recognized primarily based on monophyly in phylogenetic trees derived from chloroplast genome data. Members form single or multiple primary gonimoblasts. Carposporophytes are naked or with an unconsolidated involucre of filaments. Four families, Nemaliaceae, Liagoraceae, Yamadaellaceae, and Liagoropsidaceae, are included. The number of gonimoblast initials and cell division orientation of zygotes are the most important morphological criteria for separating the families in the suborder. The morphological features used for separating genera mainly including a combination of thallus whether or not calcified, whether or not involucral/sterile filaments intermixing with gonimoblasts, involucral/sterile filaments morphologies and where they being produced, whether or not cells of carpogonial branch fused, morphologies of carposporophytes and carposporangia.

### Genera included in suborder

Nemaliaceae

- ***Nemalion*** **Duby (type genus)**

Liagoraceae

- *Akalaphycus* Huisman, I.A. Abbott & A. R. Sherwood
- *Cumagloia* Setchell & N. L. Gardner
- *Cylindraxis* Kraft
- *Dermonema* Harvey ex Heydrich
- *Dotyophycus* I. A. Abbott
- *Ganonema* K. C. Fan & Yung-C. Wang
- *Gloiocalis* S.-M. Lin, Huisman & D. L. Ballantine
- *Gloiotrichus* Huisman & Kraft
- *Helminthocladia* Agardh
- *Helminthora* Agardh
- *Hommersandiophycus* S.- M. Lin & Huisman
- *Izziella* Doty
- *Liagora* Lamouroux
- *Macrocarpus* S.-M. Lin, S. Y. Yang & Huisman
- *Neoizziella* S.-M. Lin, S. Y. Yang & Huisman
- *Patenocarpus* Yoshizaki
- *Sinocladia* C. K. Tseng & W. Li
- *Stenopeltis* Itono & Yoshizaki
- *Titanophycus* Huisman, G. W. Saunders & A. R. Sherwood
- *Trichogloea* Kützing
- *Trichogloeopsis* I. A. Abbott & Doty
- *Yoshizakia* S.-M. Lin, Huisman & C. Payri

Liagoropsidaceae

- *Liagoropsis* Yamada

Yamadaellaceae

- *Yamadaella* I. A. Abbott

### Plastid phylogenomics in red algae

This study shows that chloroplast genomes are a useful source of data to resolve phylogenetic relationships in red algae. The highly conserved nature of these genomes streamlines the assembly and annotation process. Our study, with denser sampling within a single order, along with other studies, with sparser but broader sampling, show that chloroplast genomes provide power to resolve phylogenetic relationships at all levels. We clearly demonstrated that the medium-rate and rapidly evolving plastid genes resolve phylogenetic relationships within the order for alignments as short as 2500 bp, while slow genes had much poorer performance. While the medium and fast evolving genes reached 100 bootstrap values for all but one node (Additional file 3), the slow genes only recovered 70 % of the nodes with full support. Two of these nodes had a bootstrap value lower than 60, which would be considered as inconclusive in molecular phylogenies. The choice of these slow evolving markers made sense in the early days of molecular phylogenetics when data generation was dependent on PCR amplification but in this HTS era we have easy access to more informative genes. Even for projects with limited budgets, employing HTS to obtain lower-coverage data from plastid genomes would seem like a reasonable strategy. While such data may not permit complete assembly of the genomes, the gene data derived from smaller contigs can be extracted and used in phylogenetic analysis. Considering that our analyses of subsamples of data yielded great support for alignments > 2500 nt, incompleteness of the datasets seems unlikely to result in major decreases in phylogenetic support.

We should highlight that 17 of our 22 taxa were pooled with a green algae species prior to library preparation and we were still able to confidently assembly entire plastids for most of them. This reflects an obvious lower cost per sample with similar outcomes. However, we did notice that high quality DNA is required when using HTS techniques (ratios of ~1.8 and ~2.0-2.2 for absorbance between 260 nm and 280 nm, and 260 nm and 230 nm, respectively). The major difficulty in isolating high quality DNA in algae is related to the level of polysaccharides and polyphenols present in the tissue [61]. If not totally removed these compounds can interfere and inhibit downstream applications [62]. We found that our adapted CTAB protocol resulted in good quality genomic DNA for HTS purposes in most of our target species but experiments with other red algae indicate that this cannot be generalized (unpublished results), and it is worthwhile to carry out trials prior to large-scale HTS projects [63].

It is also worth noting that while our study focused on chloroplast DNA, total genomic DNA was sequenced, yielding useful additional data from the mitochondrion and nucleus that can be used for phylogenetics. This means that from the same HTS data many other questions can be addressed with no extra cost and limited extra labour.

## Conclusions

We showed that chloroplast phylogenomics is an attractive approach for phylogenetic studies in red algae: not only because they are highly conserved, and thus straightforward to work with, but also because their plastids contain enough signal to solve phylogenetic relationships across taxonomic levels. Moreover considering the key role of red plastids in the emergence of the eukaryotic cell, the generation of more HTS data for distinct red algal groups can shed light on eukaryote evolution.

## Additional files

Additional file 1: Summary table of sequencing and assembly information per sample. Illumina platform used for each sample is discriminated with the correspondent length of individual reads in brackets. Number of paired-end reads (PE reads) generated for each sample in millions (M). Average, minimum and maximum coverage per contig is shown. (XLSX 44 kb)
Additional file 2: Details of individual evolutionary relative rates per gene: slow category (<0.75), medium category (0.75-1.5) and fast category (>1.5). All genes are represented but for further analysis only alignments ≥ 800 bp were considered. (XLSX 41 kb)
Additional file 3: Maximum likelihood aa trees estimated for the different gene rate categories showing full support for every node for fast category, a single node < 100 bootstrap for the medium and weak support for multiple nodes in the slow rate category. (PDF 841 kb)

## Abbreviations

Aa: Amino acid
CDS: Coding DNA sequence
cp: Chloroplast
CTAB: Cetyltrimethylammonium bromide
HTS: High-throughput sequencing
ML: Maximum likelihood
nt: Nucleotide
