# Supplementary figures and images for: Chloroplast genomes as a tool to resolve red algal phylogenies: a case study in the Nemaliales
Source: BMC Evol Biol. 2016 Oct 10;16:205. doi: 10.1186/s12862-016-0772-3 (PMC5057469; doi:10.1186/s12862-016-0772-3)

ML TREE FOR SLOW RATE CATEGORY

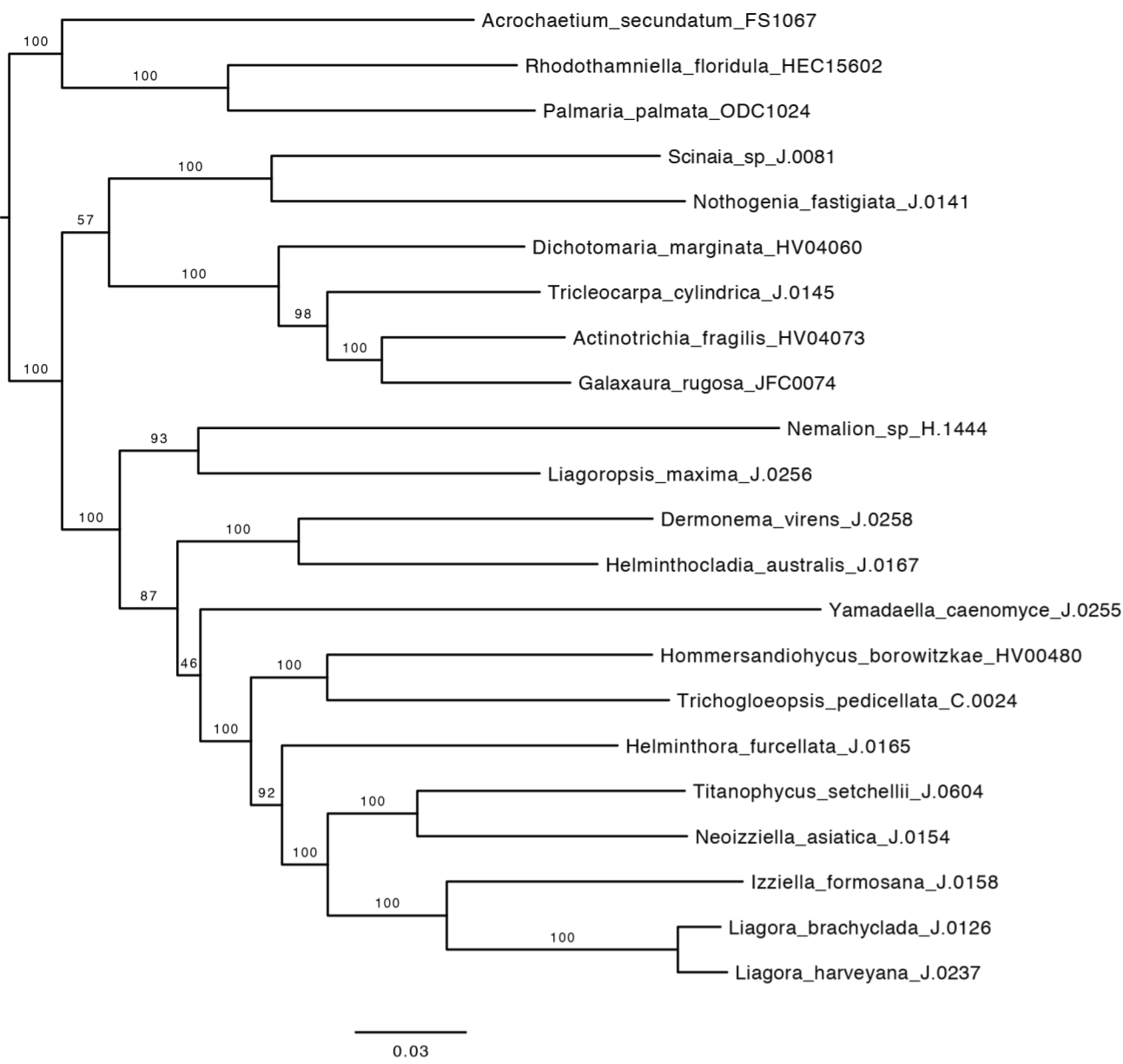

ML TREE FOR MEDIUM RATE CATEGORY

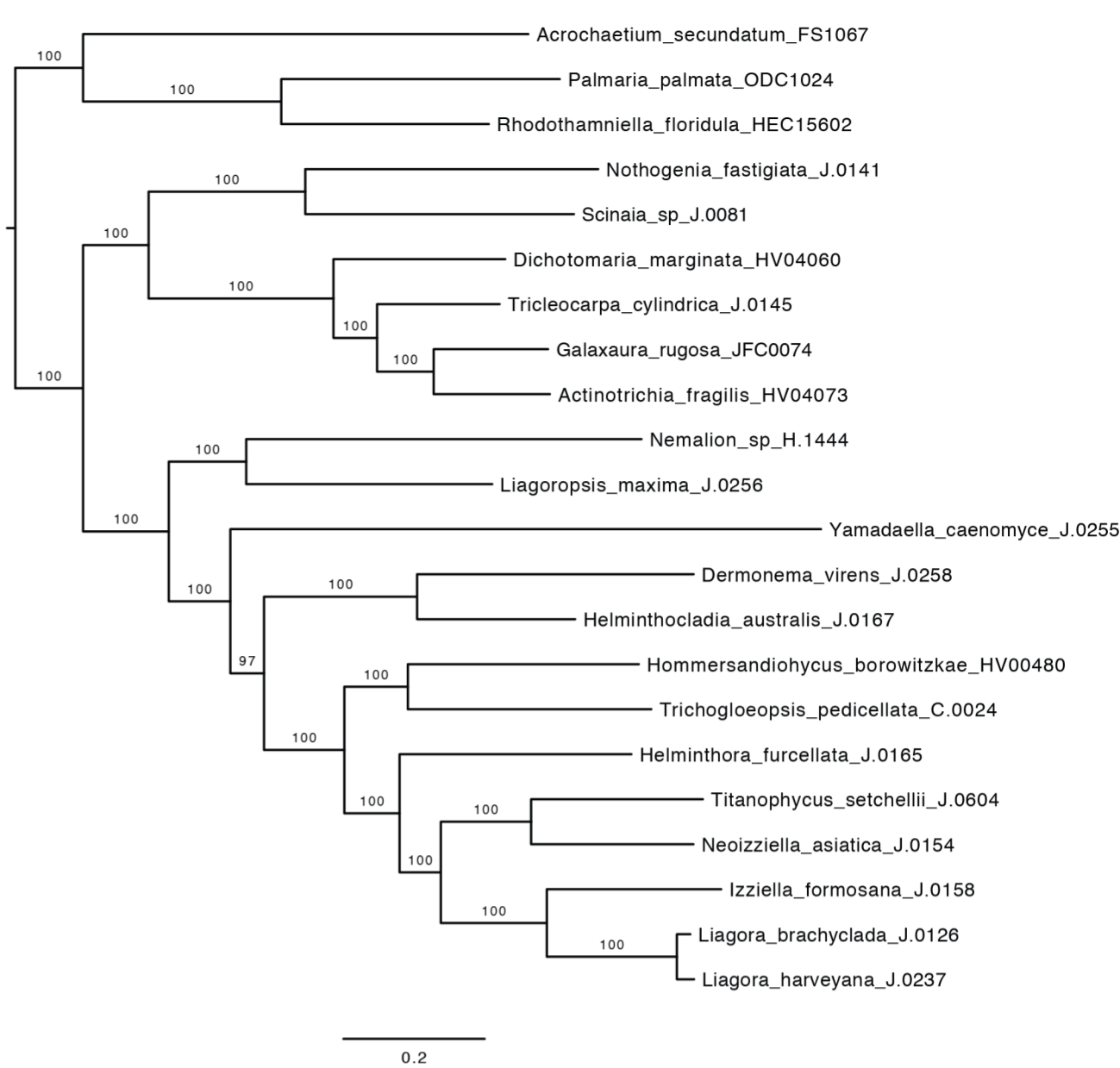

ML TREE FOR FAST RATE CATEGORY

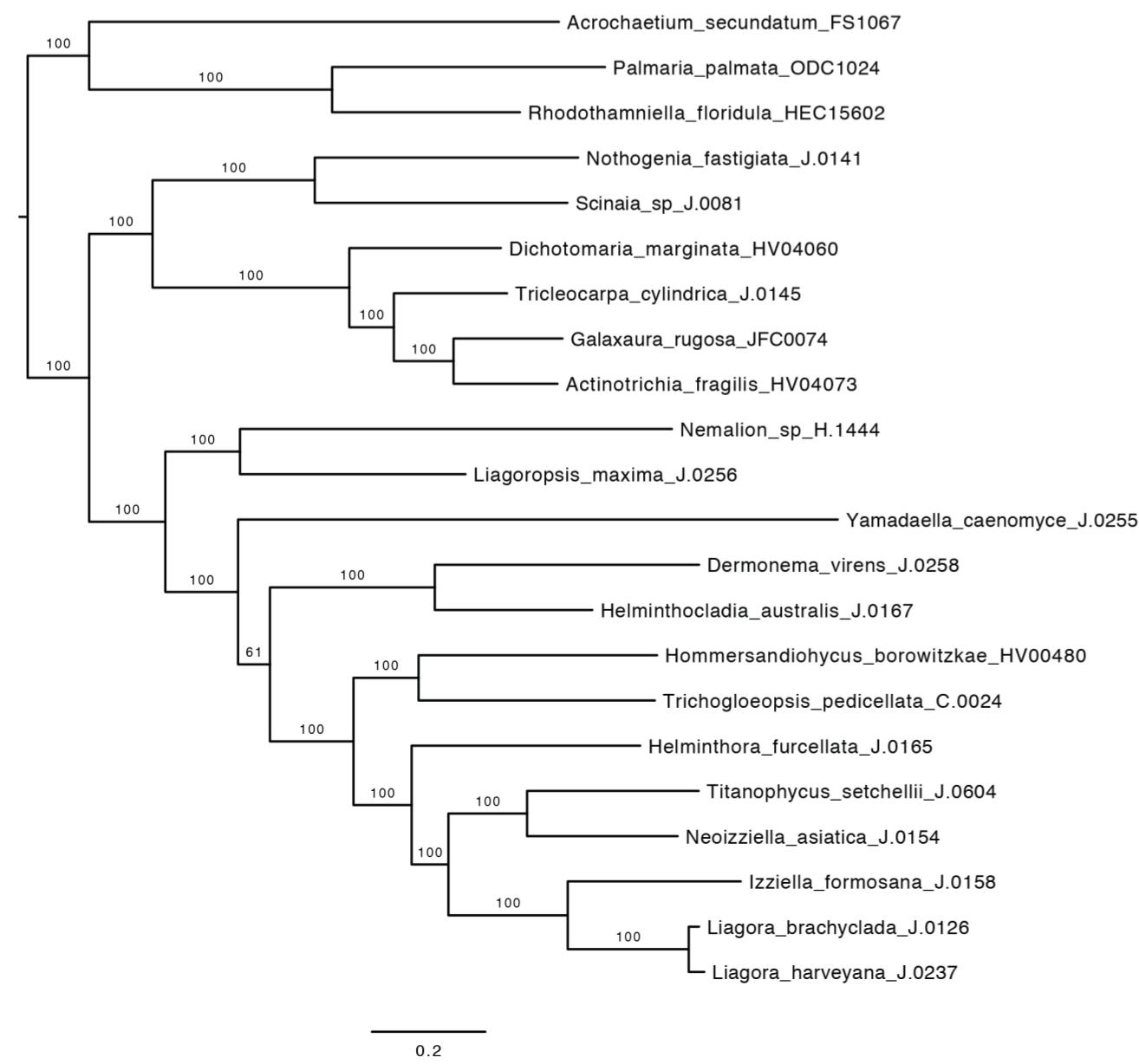

Supplement: Additional file 3: — Maximum likelihood aa trees estimated for the different gene rate categories showing full support for every node for fast category, a single node < 100 bootstrap for the medium and weak support for multiple nodes in the slow rate category. (PDF 841 kb) [file 12862_2016_772_MOESM3_ESM.pdf]
